# Plasma exchange aids thyroidectomy in refractory amiodarone-induced thyrotoxicosis, despite variable biochemical responses

**DOI:** 10.1530/ETJ-25-0351

**Published:** 2026-03-16

**Authors:** Michelle Maher, Robert McEvoy, Vanessa Farnan, Sarah Lawless, David J Tansey, Susan McKenna, Rory McQuillan, Steven Frohlich, Margaret Griffin, Jonathan Lyne, Colm Magee, Carol Traynor, Amy Hudson, Neville P Shine, James Paul O’Neill, David Halsall, Michael W O’Reilly, Amar Agha, Mark Sherlock, Carla Moran

**Affiliations:** ^1^ Department of Endocrinology, Beaumont Hospital, Dublin, Ireland; ^2^ Department of Endocrinology, St Vincent’s University Hospital, Dublin, Ireland; ^3^ Endocrine Section, Beacon Hospital, Sandyford, Dublin, Ireland; ^4^ General Internal Medicine Section, Beacon Hospital, Sandyford, Dublin, Ireland; ^5^ Critical Care Partnership, Beacon Hospital, Sandyford, Dublin, Ireland; ^6^ School of Medicine, University College Dublin, Dublin, Ireland; ^7^ Cardiology Section, Beacon Hospital, Sandyford, Dublin, Ireland; ^8^ Department of Nephrology, Beaumont Hospital, Dublin, Ireland; ^9^ Surgery Section, Beacon Hospital, Dublin, Ireland; ^10^ Department of Otorhinolaryngology, Head and Neck Surgery, Beaumont Hospital, Dublin, Ireland; ^11^ Department of Otorhinolaryngology, Head and Neck Surgery, Royal College of Surgeons in Ireland (RCSI), Dublin, Ireland; ^12^ Department of Clinical Biochemistry, Cambridge University Hospitals NHS Foundation Trust, Cambridge, UK; ^13^ Department of Medicine, Royal College of Surgeons in Ireland (RCSI), University of Medicine and Health Sciences, Dublin, Ireland

**Keywords:** amiodarone-induced thyrotoxicosis, refractory thyrotoxicosis, plasma exchange, plasmaphaeresis

## Abstract

**Introduction:**

Amiodarone-induced thyrotoxicosis (AIT) is a serious complication of amiodarone therapy, associated with high morbidity and mortality. Standard medical therapies are often insufficient in refractory cases, and therapeutic plasma exchange (TPE) has been proposed as a bridge to definitive thyroidectomy, although protocols for its use vary and detailed descriptions and definitions of response to therapy are limited.

**Case presentation:**

We report four cases of severe refractory AIT, prepared for thyroidectomy with TPE. Patients underwent three to five sessions using either albumin with saline, or combinations including fresh frozen plasma, as replacement fluids. TPE produced variable biochemical effects: free thyroxine (FT4) levels consistently fell during sessions but rarely normalised, while total thyroxine (TT4) normalised in some cases. In most patients, thyroid-stimulating hormone (TSH), previously suppressed for months, rose to detectable levels after only one to two sessions, suggesting a rapid reduction in biologically active thyroid hormone (TH) concentration. TPE was well tolerated overall, although transient coagulopathy and thrombocytopaenia occurred in two cases. All patients proceeded to successful thyroidectomy and achieved post-operative euthyroidism.

**Conclusion:**

TPE may provide temporary biochemical improvement and clinical stabilisation in refractory AIT, facilitating safe progression to thyroidectomy. However, its biochemical effects can be inconsistent and transient, and complications such as coagulopathy must be anticipated. Our experience supports the use of TPE as a valuable adjunct in selected patients with refractory AIT but illustrates that thyroidectomy should not be unnecessarily delayed in pursuit of complete TH normalisation or rigid biochemical targets.

## Established facts

- Refractory thyrotoxicosis is uncommon but presents significant management challenges. Definitive treatment with thyroidectomy may be required; however, patients should ideally achieve a euthyroid state before surgery. Adjunctive strategies to induce euthyroidism include lithium, cholestyramine, perchlorate, iopanoic acid and therapeutic plasma exchange, although the availability of these therapies varies globally.

## Novel insights

- The biochemical response to therapeutic plasma exchange is complex. We observed initial reductions in free T4 that were transient; however, concurrent decreases in total T4 concentrations and subtle increments in TSH suggested that overall thyroid status is ameliorated.
- Despite such transient or inconsistent improvements in thyroid hormone measurements, therapeutic plasma exchange remains a helpful tool to improve the clinical status of a severely thyrotoxic patient and facilitate urgent thyroid surgery.

## Introduction

Amiodarone is an anti-arrhythmic agent that causes thyroid dysfunction in 15–20% of patients (1). The environmental iodine uptake and underlying thyroid status predispose patients to amiodarone-induced thyrotoxicosis (AIT) or amiodarone-induced hypothyroidism (2). There are two predominant forms of AIT: type 1, a form of iodine-induced hyperthyroidism in the setting of underlying thyroid dysfunction, and type 2, a destructive thyroiditis (3). Mixed and indeterminate forms also exist. First-line medical treatment for type 1 AIT is thionamide medication, whereas type 2 AIT usually responds well to glucocorticoid therapy (4, 5).

AIT is associated with significant mortality, especially in patients with a reduced ejection fraction (EF), where it reaches over 30% (6, 7). Management of AIT after conventional medical therapies fail can be very challenging. Options for rendering patients euthyroid in refractory AIT include iopanoic acid, perchlorate, cholestyramine, lithium and therapeutic plasma exchange (TPE) (5, 8, 9). There is no agreed optimal therapy in such cases, and different centres have variable access to these second-line therapies.

TPE is recommended by the American Society of Apheresis (9), Japan Endocrine Society and Japan Thyroid Association (10) as a treatment for thyroid storm (TS) in patients who respond poorly to first-line therapies. TPE is an extracorporeal blood purification technique, through which the patient’s plasma is replaced with fresh plasma, albumin or crystalloid, removing both the intravascular protein-bound TH and free TH. TPE also introduces additional unsaturated binding sites for free TH through thyroid hormone-binding proteins (THBPs), such as thyroxine-binding globulin (TBG) in plasma, or albumin (9). Putative additional beneficial effects include the removal of amiodarone from the circulation, along with factors implicated in TS, such as thyroid autoantibodies, cytokines and catecholamines (11).

No clear consensus exists regarding the role of TPE in thyrotoxicosis not associated with TS, with safety and efficacy data limited to case reports and case series. In addition, protocols for TPE (type and volume of replacement fluid administered and frequency of TPE sessions) are not firmly established. Goals of treatment are also not well described, with most reports, but not all, stating both clinical and biochemical improvements (10, 12). We report our recent experience using TPE to prepare for salvage thyroidectomy in refractory AIT.

## Case presentations

### Case 1

**Figure 1:**
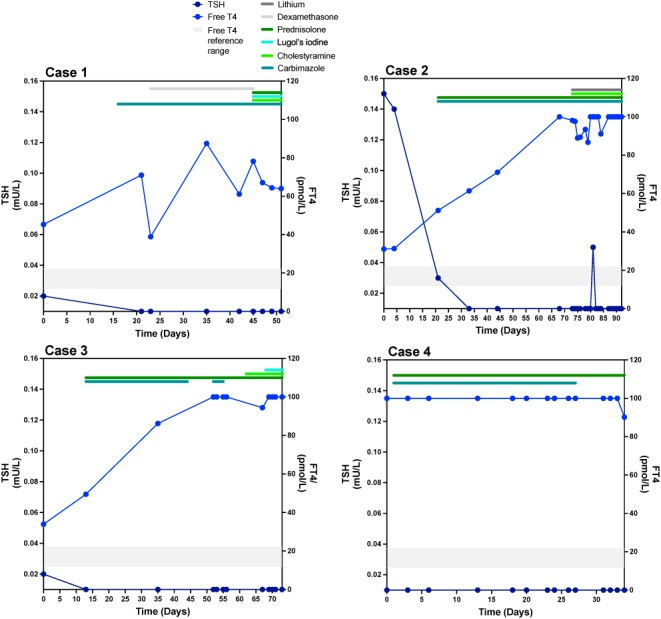
Graphs showing TSH and FT4 levels and medications used to control thyrotoxicosis prior to TPE in all four cases. The shaded grey box indicates the reference interval for FT4. Reference ranges: TSH: 0.27–4.20 mU/L; FT4: 12–22 pmol/L (Cases 1, 2 and 3) and 11.9–21.6 pmol/L (Case 4); and FT3: 2.43–6.01 pmol/L (Case 1) and 3.1–6.8 pmol/L (Cases 2, 3 and 4).

**Figure 2:**
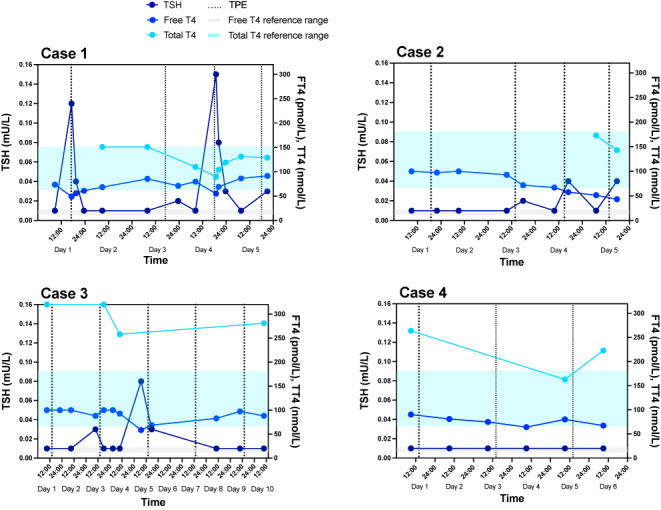
Graphs showing the biochemical response to TPE in all cases. The shaded boxes indicate the reference intervals for FT4 (grey) and TT4 (blue). The vertical dashed lines indicate days of TPE sessions. Reference ranges: TSH: 0.27–4.20 mU/L; FT4: 12–22 pmol/L (Cases 1, 2 and 3) and 11.9–21.6 pmol/L (Case 4); FT3: 2.43–6.01 pmol/L (Case 1) and 3.1–6.8 pmol/L (Cases 2, 3 and 4); and TT4: 63–151 nmol/L (Case 1) and 66–181 (Cases 2, 3 and 4).

A 53-year-old man was referred to our care with AIT, likely type 2 (TSH = 0.02 mU/L (RR: 0.27–4.2), FT4 = 45.3 pmol/L (RR: 12–22), TSH receptor Ab (TRAb): < 0.8 IU/L (RR: < 0.8)) (Supplementary Table 1 (see section on Supplementary materials given at the end of the article)). He had a significant cardiac history, with heart failure with a reduced EF in the setting of familial lamin A/C cardiomyopathy, refractory ventricular tachycardia (requiring bilateral thoracic sympathectomy and cardiac denervation), complete heart block, atrial fibrillation and cardiac defibrillator *in situ*. He became progressively more thyrotoxic despite treatment with carbimazole and dexamethasone (TSH: < 0.01 mU/L (RR: 0.27–4.2), FT4 = 61.1 pmol/L (RR: 12–22)) (Fig. 1, Supplementary Table 2), resulting in decompensated heart failure and necessitating hospital admission for intravenous diuresis. He was managed with escalating doses of carbimazole and glucocorticoids, along with the addition of aqueous iodine oral solution and cholestyramine, with no improvement in his thyroid status (TSH: < 0.01 mU/L (RR: 0.27–4.2), FT4 = 64 pmol/L (RR: 12–22); Fig. 1, Supplementary Table 2). In consultation with cardiology, we opted to continue amiodarone, given the history of ventricular tachycardia. TPE was chosen as a bridge therapy to total thyroidectomy. He underwent four sessions of TPE over five days, with FFP and 5% albumin as replacement fluids (Table 1, Fig. 2). Thyroid function tests (TFTs) during TPE demonstrated a reduction in FT4 concentration and a rise in TSH within one hour of TPE (for example, day 4, one hour following TPE session 3 – TSH = 0.15 mU/L (RR: 0.27–4.2), FT4 = 55 pmol/L (RR: 12–22), TT4 = 90 nmol/L (RR: 63–151)); however, these biochemical improvements were not sustained (day 5, prior to TPE session 4 – TSH = 0.01 mU/L (RR: 0.27–4.2), FT4 = 86.5 mU/L (RR: 12–22)). Notably, TT4 normalised (129 nmol/L, RR: 63–151), despite markedly elevated FT4 = 91.5 pmol/L (RR: 12–22) (Table 1, Fig. 2). He tolerated TPE well; however, he developed thrombocytopaenia, so heparin was omitted on the final TPE session. Thyroidectomy was undertaken one day following the fourth TPE session and was uneventful, with a stable intra-operative course. He was discharged home on levothyroxine and subsequently rendered euthyroid. Unfortunately, he died 10 months later following further deterioration of his cardiac status, despite maintenance of euthyroidism.

**Table 1:** Detailed biochemical response to TPE in all cases. TPE regimens were as follows – Case 1: 4 sessions over 5 days, 1 PV replaced by 2 units of fresh frozen plasma and 5% albumin; Case 2: 4 sessions over 5 days; Case 3: 5 sessions over 9 days (performed on alternate days); Case 4: 3 sessions over 5 days (performed on alternate days). In Cases 2, 3 and 4, 1 plasma volume was replaced by 5% albumin (two-thirds) and 0.9% saline (one-third).

Case/time	TSH (mU/L)	FT4 (pmol/L)	FT3 (pmol/L)	TT4 (nmol/L)
Case 1				
Day 1: TPE1				
Pre-TPE	<0.01	73.6	5.69	-
TPE1 + 1 h	0.12	49.1	-	-
TPE1 + 2 h	0.04	55.9	-	-
TPE1 + 6 h	0.01	61	-	-
Day 2	0.01	68.5	6.62	151
Day 3: TPE2				
Pre-TPE2	<0.01	85.4	7.73	151
TPE2 + 6 h	0.02	71.1	-	-
Day 4: TPE3				
Pre-TPE3	0.01	79.7	7.93	110
TPE3 + 1 h	0.15	55	7.79	90
TPE3 + 2 h	0.08	69.1	8.45	104
TPE3 + 5 h	0.03	-	-	119
Day 5: TPE4				
Pre-TPE4	0.01	86.5	10.34	131
TPE4 + 3 h	0.03	91.5	12.56	129
Case 2				
Day 1: TPE1				
Pre-TPE	<0.01	>100	-	-
TPE1 + 3 h	<0.01	97.5	-	-
Day 2	<0.01	>100	-	-
Day 3: TPE2				
Pre-TPE2	<0.01	93.1	-	-
TPE2 + 4 h	0.02	71.8	-	-
Day 4: TPE3				
Pre-TPE3	<0.01	67.2	-	-
TPE3 + 2 h	0.04	57.6	-	-
Day 5: TPE4				
Pre-TPE4	<0.01	51.6	10.7	173
TPE4 + 4 h	0.04	43.3	10.9	143
Case 3				
Day 1: TPE1				
Pre-TPE	<0.01	>100	-	>320
Day 2: no TPE		>100	13.5	-
Day 3: TPE2				
Pre-TPE2	<0.01	>100	15.1	-
TPE2 + 5 h	0.03	88.0	11.7	-
Day 4	<0.01	>100	13.3	320
Day 5: TPE3	<0.01	>100	14.1	-
Day 6	<0.01	92.6	-	258
Day 7: TPE4				
TPE4 + 3 h	0.08	58.4	-	-
TPE4 + 4 h	0.03	68.7	9.2	-
Day 8	0.01	82.8	-	-
Day 9: TPE5				
Pre-TPE5	<0.01	97.2	-	-
TPE5 + 19 h	<0.01	88.1	13.1	281
Case 4				
Day 1: pre-TPE1	<0.01	90.2	8.2	264
Day 2	<0.01	81	-	-
Day 3: pre-TPE2	<0.01	74.8	-	-
Day 4	<0.01	64	-	-
Day 5: pre-TPE3	<0.01	80.1	-	163
Day 6	<0.01	67.3	-	223

Reference ranges: TSH: 0.27–4.20 IU/mL; FT4: 12–22 pmol/L (Cases 1, 2 and 3) and 11.9–21.6 pmol/L (Case 4); FT3: 2.43–6.01 pmol/L (Case 1) and 3.1–6.8 pmol/L (Cases 2, 3 and 4); and TT4: 63–151 nmol/L (Case 1) and 66–181 (Cases 2, 3 and 4).

TPE, therapeutic plasma exchange.

### Case 2

A 47-year-old man was referred to our services following identification of thyrotoxicosis on routine blood testing (TSH = 0.15 mU/L (RR: 0.27–4.2), FT4 = 31.1 pmol/L (RR: 12–22)) (Supplementary Table 1). He had a history of atrial fibrillation treated with amiodarone but had stopped taking it for 5 months prior to presentation following a successful ablative procedure. His past medical history included tachycardiomyopathy, type 2 diabetes mellitus, hypertension and asthma. Treatment with carbimazole and prednisolone was initiated prior to review in our clinic. He remained clinically and biochemically thyrotoxic despite first-line treatments and was admitted to hospital for optimisation of thyrotoxicosis and recurrent fast atrial fibrillation. Second-line therapies, including cholestyramine and lithium, had no effect (TSH < 0.01 mU/L (RR: 0.27–4.2), FT4 > 100 pmol/L (RR: 12–22)) (Fig. 1, Supplementary Table 2). The patient underwent four sessions of TPE over five days, with 5% albumin and 0.9% saline as replacement fluids (ratio 2:1) (Table 1, Fig. 2). Following TPE sessions, TFTs showed a reduction in FT4 with a marginal rise in TSH within hours (day 3, four hours following TPE session 2 – TSH = 0.02 mU/L (RR: 0.27–4.2), FT4 = 71.8 pmol/L (RR: 12–22)), with FT4 reduction sustained by day 5 (TSH = 0.04 mU/L (RR: 0.27–4.2), FT4 = 43.3 pmol/L (RR: 12–22)). Again, TT4 was within the reference range (143 nmol/L, RR: 66–181), despite contemporaneously raised FT4 concentrations (43.3 pmol/L, RR: 12–22) (Table 1, Fig. 2). TPE was complicated by coagulopathy with hypofibrinogenaemia and thrombocytopaenia, requiring vitamin K and fibrinogen replacement. Thyroidectomy was performed one day following the fourth TPE session. The intra-operative course was stable; however, atrial fibrillation with a fast ventricular rate recurred within hours post-operatively (TSH < 0.01 mU/L (RR: 0.27–4.2), FT4 = 42.9 pmol/L (RR: 12–22)), necessitating additional rate control in the coronary care unit, but the patient subsequently reverted to sinus rhythm three days later. He was later rendered euthyroid with levothyroxine and has remained well.

### Case 3

A 56-year-old woman presented with thyrotoxicosis, while on amiodarone for ventricular fibrillation with associated dilated cardiomyopathy and heart failure with a reduced EF. Baseline investigations were suggestive of AIT type 2 (TSH = 0.02 mU/L (RR: 0.27–4.2), FT4 = 33.9 pmol/L (RR: 12–22), TRAb < 0.8 IU/L (RR: < 0.8 IU/L); Supplementary Table 1). Amiodarone was stopped at diagnosis of AIT. She became rapidly and progressively more thyrotoxic, despite treatment with carbimazole (later stopped due to hepatotoxicity), prednisolone, aqueous iodine oral solution and cholestyramine (TSH < 0.01 mU/L (RR: 0.27–4.2), FT4 > 100 pmol/L (RR: 12–22)) (Fig. 1, Supplementary Table 2) and so proceeded to thyroidectomy with pre-operative TPE. She underwent five TPE sessions over nine days, with 5% albumin and 0.9% saline as replacement fluids (ratio 2:1). Following TPE, a small reduction in FT4, with a rise in TSH, was observed (for example, day 7, four hours following TPE session 4 – TSH = 0.03 mU/L (RR: 0.27–4.2), FT4 = 68.7 pmol/L (RR: 12–22)), with some reduction in FT4 being sustained by day 9 (FT4: 88.1 pmol/L). TT4 remained elevated but reduced from baseline (day 1: >320 nmol/L; day 9: 281 nmol/L, RR: 66–181) (Table 1, Fig. 2). The patient proceeded to uncomplicated thyroidectomy two days following the fifth TPE treatment, with a stable intra-operative course. She is euthyroid on levothyroxine and clinically well.

### Case 4

A 65-year-old man was admitted from cardiology clinic for management of atrial fibrillation with a rapid ventricular response. His background history included heart failure with a reduced EF (35–40%). He had known atrial fibrillation with an unsuccessful DC cardioversion 2 years previously, after which he had been commenced on amiodarone. Investigations supported a diagnosis of AIT type 2 (TSH < 0.01 mU/L (RR: 0.27–4.2), FT4 > 100 pmol/L (RR: 11.9–21.6), TRAb < 0.8 IU/L, RR: < 0.8)) (Supplementary Table 1). After three weeks of treatment with high-dose prednisolone and carbimazole, he remained thyrotoxic (TSH <0.01 mU/L (RR: 0.27–4.2), FT4 = 90.2 pmol/L (RR: 11.9–21.6), TT4 = 264 nmol/L (RR: 66–181)) (Fig. 1, Supplementary Table 2). An echocardiogram showed evolving severe left ventricular systolic dysfunction (EF < 30%), prompting a decision to proceed to urgent thyroidectomy with pre-operative TPE. The patient underwent three TPE sessions over five days, with 5% albumin and 0.9% saline as replacement fluids (ratio 2:1). A reduction in both FT4 (day 4: 64 pmol, RR: 11.9–21.6) and TT4 (day 5: 163 nmol/L, RR: 66–181) was observed, but no change in TSH (Table 1, Fig. 2). The patient underwent an uncomplicated thyroidectomy one day following the third TPE session, with a stable intra-operative course. Post-operatively, his atrial fibrillation remained difficult to control and he underwent another unsuccessful electrical cardioversion day 7 post-operatively (FT4 = 16.4 pmol/L, RR: 11.9–21.6). He was discharged on sotalol and levothyroxine. He awaits pulmonary vein isolation for definitive management of his atrial fibrillation.

## Discussion

Thyrotoxicosis that is resistant to standard treatments (here termed ‘refractory’) is rare, but very difficult to manage. While it can arise due to many aetiologies, AIT is a frequent cause (13). Patients with AIT typically have additional comorbidities that reduce their tolerability to the thyrotoxic state and increase the associated morbidity and mortality. In patients with refractory thyrotoxicosis, definitive management in the form of thyroidectomy is ideal, but restoration of euthyroidism prior to this can be challenging. There is no universal preferred method of achieving euthyroidism in refractory thyrotoxicosis, and access to and experience of these second-line therapies vary throughout the world.

In our institutions, we do not have access to perchlorate or iopanoic acid and so have used TPE as a bridge to salvage thyroidectomy in refractory thyrotoxicosis. Here, we describe the biochemical response and clinical outcomes following TPE in four patients with uncontrolled, severe, AIT that was clearly unresponsive to standard treatments.

We observed a variable biochemical response to TPE in thyrotoxicosis. All our cases showed some reduction in FT4 levels during TPE, but none normalised and, in most, the reduction was not sustained. Interestingly, despite TSH suppression in all cases for 1–3 months prior to TPE, TSH concentrations increased to detectable levels after only one or two TPE sessions in 3 of 4 cases, suggesting a rapid and profound reduction in circulating free TH concentrations. Considering the robustness of TSH measurements, even this small change likely indicates a reduction in thyroid hormone action on pituitary thyrotrophs. Total thyroid hormone measurements, which are dominated by protein-bound rather than ‘free’ thyroid hormone, were also available for some samples, and this parameter normalised following TPE in two of the cases.

The current literature shows that although the majority of thyrotoxic patients achieve a reduction in FT4 levels following TPE (12, 14, 15), many exhibit variable levels of FT4 reduction (12, 14, 16), which is often transient (17, 18, 19). Similar to two of our cases, others have observed a subtle increase in TSH on TPE, despite persistently elevated FT4 levels (16, 18, 20, 21, 22, 23, 24, 25, 26). TT4 levels are infrequently reported but usually fall or normalise (18, 27); similar to our cases, others have also reported normalisation of TT4 despite persistently elevated FT4 levels (18).

Interpretation of thyroid hormone concentrations following TPE is challenging given that the vast majority (>99%) of circulating thyroid hormone is tightly protein bound. The combination of high-affinity (TBG) and high-capacity, low-affinity (albumin) T4-binding sites ensures that, *in vivo*, circulating free thyroid hormone concentration remains constant despite transient fluctuations in T4 production. In the context of TPE, FT4 concentration will be maintained despite removal of large amounts of protein-bound T4 if ‘empty’ thyroid hormone-binding sites are not replaced in the exchange fluid, as the T4-binding equilibrium will be quickly re-established with the residual binding proteins (11). For this reason, FFP has been proposed as a better exchange fluid than albumin due to the presence of higher-affinity TBG-binding sites (28). Here, we used FFP and albumin in one case and albumin with saline in three cases, so there were too few cases to allow us to discern whether our choice of replacement fluid affected the biochemical outcome. A scoping review of over 200 cases in which TPE was employed to manage thyrotoxicosis prior to thyroidectomy found no difference in biochemical outcomes based on the type of replacement fluid used (28).

One previous report demonstrated that TPE modifies extravascular (as well as intravascular) thyroid hormone concentrations and may do so at variable rates across treatment sessions, resulting in redistribution of TH from tissue stores to the intravascular compartment. It is possible that such fluctuations could also affect the measured free fraction of circulating TH (29). Given the fluidity of free thyroid hormone measurements, total thyroid hormone is likely to be a better marker than FT4 for treatment efficacy (defined as amount of T4 that has been removed), although TT4 may not reflect contemporary thyroid status if thyroid hormone-binding proteins have not been replaced.

There may also be analytical issues with measuring FT4 in the context of TPE, which may further confound TFT interpretation. FT4 assays are exquisitely sensitive to analytical conditions and are designed to provide accurate results in typical human plasma. TPE may affect clinical free hormone assays by altering the concentration of thyroid hormone-binding proteins and by changing the complex milieux of other possible competitors for thyroid hormone-binding protein sites in plasma. The effect of heparin, used to prevent clotting in the extracorporeal circuit during TPE (30), on free thyroid hormone assays is well described. Heparin is known to activate endothelial lipase, releasing free fatty acids and displacing TH from THBPs *in vitro*; thus, the presence of heparin in a serum sample can artefactually elevate free TH level (31). Notably, total TH levels remain unaffected by heparin administration and are therefore the preferred measure for evaluating TH status in the presence of heparin.

The aetiology of thyrotoxicosis likely also influences response to TPE, with those who demonstrate early rises in FT4 following TPE and inability to maintain TH reduction likely having very active thyrotoxicosis, associated with pronounced and sustained TH release from the thyroid gland. In addition, thyrotoxicosis mediated by TRAb may respond better to TPE, since TPE also effectively removes circulating TRAb (10, 17, 29).

It is possible that the variable biochemical response we observed is related to the operational procedures for TPE (frequency and number of sessions and replacement fluids); however, we used similar procedures to that described in the literature and recommended by the American Society for Apheresis (9) and our centres are experienced in using this technique, making procedural errors unlikely.

Clinical response to TPE is difficult to ascertain in an acutely unwell patient, particularly when there is coexistent cardiac disease. Standard assessments of thyroid status, such as thyrotoxic symptoms, heart rate and rhythm, temperature and agitation, can all be confounded by coexistent cardiac disease (arrhythmia and heart failure) or additional complications that can arise in acutely unwell patients (infection and sepsis). In addition, frequent adjustments in elements of supportive care (use of beta blockers, cooling agents and antibiotics) may alter these parameters independently of changes in TH concentrations. Acknowledging these limitations, all our patients remained clinically stable, without evidence of decompensation of their cardiac or thyrotoxic state while on TPE.

TPE appears generally safe in the management of thyrotoxicosis; however, several important considerations should be noted. TPE can reduce the plasma concentration of clinically important medications, such as propranolol and certain antibiotics (e.g. gentamicin); therefore, consultation with a clinical pharmacist is recommended before commencing TPE (32). TPE-induced coagulopathy is a known complication and is of particular relevance to this patient population in whom atrial fibrillation, and hence a requirement for therapeutic anticoagulation, is common. To negate the risk of intra-operative bleeding, some recommend the use of FFP during the final session of TPE before thyroidectomy (17, 33) and deferring surgery for 48 h after TPE (33); however, this advice should be balanced against the transient effect of TPE on TH seen in our patients and others (17, 28). With our protocol, no instances of post-operative bleeding were noted. It must be acknowledged that TPE is time-consuming, is costly and requires specialist equipment and trained staff.

TPE was well tolerated by our patients with only transient and manageable complications. All patients underwent successful thyroidectomy. Two of our patients experienced ongoing atrial fibrillation post-operatively, one of whom had persistent atrial fibrillation for over a week following restoration of euthyroidism with thyroidectomy, so we suspect that this was due to persistence of known, pre-existing atrial fibrillation, rather than exacerbation of a thyrotoxic state.

Given the complicating factors discussed above, identifying the most appropriate determinant of TPE efficacy is challenging and may involve a combination of free thyroid hormone concentrations, total thyroid hormone concentrations, clinical thyroid status and surgical outcomes. In our experience, while the clinical outcomes following urgent thyroidectomy for AIT with prior TPE use were good, the biochemical responses to TPE were variable and inconsistent. Thus, while TPE can aid in pre-surgical optimisation, a poor biochemical response should not delay definitive surgical management.

It is likely that a ‘therapeutic window’ exists for thyroidectomy in patients with refractory thyrotoxicosis. Although surgery is ideally undertaken after improvement in thyroid hormone status, prolonged delays in pursuit of euthyroidism may result in further clinical deterioration and increased operative risk. Accordingly, clinicians should recognise when additional attempts to achieve euthyroidism are unlikely to be successful and proceed with surgery despite ongoing thyrotoxicosis.

In that regard, although original reports of urgent thyroidectomy in AIT demonstrated high complication rates and mortality (34), more recent reports suggest increased safety of surgery in this setting, particularly when performed by skilled surgeons and anaesthetists (35, 36). A recent meta-analysis suggests that thyroid surgery without prior restoration of euthyroidism may not be as unsafe as once thought (37). Improvements in anaesthetic techniques and intensive care are likely to contribute to better outcomes following urgent thyroidectomy, and the favourable results observed in our cohort may reflect these advances. Caution is required in extrapolating these findings to all patients, particularly those with significant cardiac disease or those not managed in centres with experienced multidisciplinary teams, including specialised anaesthetic and surgical care. The available data are retrospective, likely subject to reporting bias and currently insufficient to support the routine and safe performance of thyroidectomy in uncontrolled thyrotoxicosis in all cases.

Ultimately, our experience demonstrates that the clinical improvement observed during TPE may not necessarily correlate with a significant reduction in circulating free TH levels, and normalisation of TH levels may not be required to achieve successful thyroidectomy.

## Conclusion

TPE usually provides biochemical improvements and clinical stabilisation in refractory AIT, facilitating safer progression to thyroidectomy. Its biochemical effects, however, can be inconsistent and transient. Our experience supports the use of TPE as a valuable adjunct in patients with refractory AIT who require thyroidectomy and suggests that measurement of TT4 and close observation of subtle changes in TSH may be helpful additional markers of TPE efficacy. Thyroidectomy should not be unnecessarily delayed in pursuit of specific biochemical targets or expectation of complete normalisation of TH concentrations by TPE.

## Supplementary materials



## Declaration of interest

CM performs honorary consultancy work for Egetis Pharmaceuticals, has received publication fees from Institut Biochimique SA (IBSA) and acts on an ad hoc basis as an expert advisor to the Health Products Regulatory Agency of Ireland. The other authors have no conflicts of interest to declare.

## Funding

This research did not receive any specific graft from any funding agency in the public, commercial or not-for-profit sector.

## Patient consent

Written informed consent for publication of clinical details was obtained from patients who may potentially be identified from the case descriptions.

## Author contribution statement

MM led the collection and assembly of the data and drafted the first version of manuscript. RMcE contributed to data collection and writing of the manuscript. VF, SL and DJT contributed to data collection. SM, RMcQ, SF, MG, JL, CMa, CT, AH, NS, JPO’N, MOR, AA, MS and CMo provided clinical care to the patients. DH provided additional biochemical analyses and interpreted the results. CMo supervised the project, revised the first version of the manuscript and prepared the final version of the manuscript. All authors revised manuscript drafts and approved the final submitted version.

## Statement of ethics

This research was conducted ethically in accordance with the World Medical Association Declaration of Helsinki. No formal ethical approval was required, since all investigations and care provided were performed during the course of routine clinical care.
