# Supplementary material for: Plasma exchange aids thyroidectomy in refractory amiodarone-induced thyrotoxicosis, despite variable biochemical responses
Source: Eur Thyroid J. 2026 Mar 16;15(2):ETJ250351. doi: 10.1530/ETJ-25-0351 (PMC13010327; doi:10.1530/ETJ-25-0351)
Supplement: Supplementary file 1 [file supplementary_materials.pdf]

## Supplementary Table 1

| Case | TSH (mU/L) | FT4 (pmol/L) | TRAb (IU/L) | Imaging |
|---|---|---|---|---|
| 1 | 0.02 (RR 0.27-4.20) | 45.3 (RR 12-22) | <0.8 (RR <0.8) | US: Normal sized thyroid gland with heterogenous echogenicity. Reduced vascularity. |
| 2 | 0.15 (RR 0.27-4.20) | 31.1 (RR 12-22) | <0.8 (RR <0.8) | US: Normal sized thyroid gland with heterogenous echogenicity. Reduced vascularity. |
| 3 | 0.02 (RR 0.27-4.20) | 33.9 (RR 12-22) | <0.8 (RR <0.8) | US: Normal sized thyroid gland with normal echogenicity. Reduced vascularity. |
| 4 | <0.01 (RR 0.27-4.20) | >100 (RR 11.9-21.6) | <0.8 (RR <0.8) | US: Slightly enlarged thyroid gland with heterogenous echogenicity. Reduced vascularity. |

**Supplementary Table 1:** Baseline biochemistry and radiology in all cases.
*Abbreviations: TRAb: TSH receptor antibody; US: ultrasound; RR: Reference range*

**Supplementary Table 2**

| | | Medication | Biochemical Results | | |
|---|---|---|---|---|---|
| **Case 1** | **Day** | | **TSH (mU/L)** | **FT4 (pmol/L)** | **FT3 (pmol/L)** |
| | 0 | - | 0.02 | 45.3 | - |
| | 21 | Carbimazole 10mg BD | <0.01 | 71.0 | 10.7 |
| | 35 | Carbimazole 20mg BD, Dexamethasone 0.5mg BD | <0.01 | 87.5 | 7.6 |
| | 42 | Carbimazole 20mg TDS, Dexamethasone 0.5mg BD | <0.01 | 61.1 | 8.0 |
| | 47 | Carbimazole 30mg TDS, Prednisolone 30mg 0D, Aqueous iodine oral solution 0.3mls TDS, Cholestyramine 16g/day | <0.01 | 67.1 | 5.56 |
| | 51 | Carbimazole 30mg TDS, Prednisolone 30mg 0D, Aqueous iodine oral solution 0.3mls TDS, Cholestyramine 16g/day | <0.01 | 64.0 | - |
| **Case 2** | 0 | - | 0.15 | 31.1 | - |
| | 21 | - | 0.03 | 51.2 | - |
| | 33 | Carbimazole 20mg OD, Prednisolone 20mg OD | 0.01 | 61.4 | 12.0 |
| | 68 | Carbimazole 20mg BD, Prednisolone 40mg OD | <0.01 | >100 | 22.6 |
| | 75 | Carbimazole 30mg BD, Prednisolone 40mg OD, Lithium 200mg BD, Cholestyramine 4g BD | <0.01 | 88.9 | - |
| | 82 | Carbimazole 30mg BD, Prednisolone 40mg OD, Lithium 400mg BD, Cholestyramine 4g TDS | <0.01 | >100 | 17.3 |
| | 92 | Carbimazole 30mg BD, Prednisolone 40mg OD, Lithium 400mg QDS, Cholestyramine 4g TDS | <0.01 | >100 | - |

| | | | | | |
|---|---|---|---|---|---|
| **Case 3** | 0 | - | 0.02 | 33.0 | - |
| | 35 | Prednisolone 40mg OD, Carbimazole 20mg TDS | <0.01 | 86.2 | 14.5 |
| | 52 | Prednisolone 30mg  OD | <0.01 | 100 | - |
| | 55 | Prednisolone 60mg OD,  Carbimazole 20mg TDS | <0.01 | >100 | 20.3 |
| | 63 | Prednisolone 40mg OD | <0.01 | >100 | - |
| | 67 | Prednisolone 60mg OD, Cholestyramine 4g TDS | <0.01 | 94.4 | 11.4 |
| | 73 | Prednisolone 20mg OD, Cholestyramine 4g TDS, Aqueous iodine oral solution 0.25ml TDS | <0.01 | >100 | 17.2 |
| Case 4 | 0 | - | <0.01 | >100 | 18.4 |
| | 20 | Prednisolone 40mg OD, Carbimazole 20mg BD | <0.01 | >100 | 25.1 |
| | 26 | Prednisolone 60mg OD,  Carbimazole 20mg BD | <0.01 | >100 | 14.7 |
| | 34 | Prednisolone 60mg OD, Carbimazole 20mg BD | <0.01 | 90.2 | 8.2 |

**Supplementary Table 2:** Thyroid Hormone levels from diagnosis of thyrotoxicosis to initiation of Therapeutic Plasma Exchange in all cases. Reference ranges: TSH: 0.27-4.20 mU/L; FT4: 12-22 pmol/L (cases 1, 2, 3), 11.9-21.6 pmol/L (case 4); FT3 2.43 – 6.01 pmol/L (case 1), 3.1-6.8 pmol/L (cases 2, 3, 4). *Abbreviations: OD: once daily; BD: twice daily; TDS: three times daily.*
